# Absorption of Roots of Permanent Teeth

**Published:** 1857-04

**Authors:** 


### ARTICLE VII.

### *Absorption of Roots of Permanent Teeth.*

In the Austrian Journal of Practical Medicine, Dr. Heider gives the following account of the absorption of the roots of the permanent teeth.

The cause of the absorption of the roots of the deciduous teeth at the period of the second dentition, has been the subject of many observations and numerous hypotheses. All that we as yet know, with certainty, in respect to it, is, that there exists an intimate connection between the development of the permanent teeth, and the absorption of the roots of the deciduous teeth. In what, however, this connection actually consists, is not yet clearly explained. This much, however, is certain, that, at the period of the development of the permanent teeth, their enveloping sacculi become more vascular, and come in immediate contact with the roots of the deciduous teeth, and consequently play a very important part in the absorption of the latter—nay, in all probability, are the sole agents in effecting it. A renewed examination of the surface of the deciduous teeth, at which absorption takes place, shows that this is always on the side that is inclined towards the advancing tooth; and that when not merely the compensatory tooth, but its neighbor, comes in contact with the same deciduous tooth, two perfectly distinct surfaces of absorption, corresponding to the points of contact of the new teeth, are presented by the former, showing that both the advancing teeth in contact with it had contributed to the absorption of its root. Another fact places the correctness of this explanation of the means by which the absorption of the deciduous tooth is effected beyond doubt. When the permanent tooth is not developed, or its development takes place in a wrong situation, the corresponding deciduous tooth is not shed, but keeps its place in the jaw in after life. This is often found to be the case with respect to what are known popularly as the eye teeth. The deciduous teeth are consequently shed, one after the other, just in the order in which, by the development of the corresponding permanent teeth, their roots are absorbed by the latter.

The foregoing facts have been known to dentists for some time, but much less familiar are they with the fact that the root of a sound permanent tooth may be absorbed in the same manner as the root of one of the deciduous teeth, by the abnormal development beneath it of another permanent tooth.

In my collection I have six permanent teeth, the roots of which have been in this manner entirely or partly absorbed. Five were extracted by myself, and the persons from whose mouths they were taken, remained under my observation; for one I am indebted to my esteemed colleague, N. Terzer. These teeth are divisible into two groups: the one, where the posterior root of the second inferior molar tooth was absorbed in consequence of the expansion of the crown of the adjoining wisdom tooth; the other, in which the root of the outer incisor was absorbed by the intruding crown of the so-called eye tooth. In all, the surfaces at which absorption has taken place, present precisely the same appearance as those of the deciduous teeth, when these have been cast at the usual period. Especially is this observable in the second inferior molar teeth. In one we have the commencement of the absorption process, in a concave semicircular depression on that part of the root which was in contact with one of the projections on the obliquely situated crown of the adjoining wisdom tooth; in another case, one half, and in a third, the entire root is removed by the development beneath it of the dens sapientia. In all these cases, the extraction of the affected tooth was rendered necessary by the intense pain suffered by the patients, connected in one with inflammation of the periosteum of the root, and in the two others with exposure of the nerve.

Equally characteristic is the surface at which absorption had occurred in the incisors. In one there is an oval depression on the posterior surface of the root, near the neck; and in the other two the root is entirely removed, and one of them exhibits a cavity corresponding to the point of the encroaching eye tooth. The removal of the affected tooth, which in one case was already very loose, became necessary, in order to give room for the development of the approaching eye teeth.

These observations show, conclusively, that the process by which, at the period of the second dentition, the roots of the deciduous teeth are removed, is neither specific nor restricted to the first set of teeth, but that it may be called into action in the case of the permanent teeth, and cause the removal of their roots also, and that it is dependently partly upon the structure of the dental tissue, and partly upon the increased vascularity of the outer portion of the sacculi of the approaching teeth. The process has great similarity to that which occasions the absorption of the bones in consequence of the development of tumors in contact with them, and the two are probably identical.—*American Journal Medical Science.*

